# *Legionella* detection and enumeration in water samples by ISO 11731-2017: which method is the most sensitive?

**DOI:** 10.1128/aem.01147-25

**Published:** 2025-10-07

**Authors:** Bruno Grandbastien, Florian Mauffrey, Gilbert Greub, Dominique S. Blanc

**Affiliations:** 1 Infection Prevention and Control Unit, Infectious Diseases Service, Lausanne University Hospital and University of Lausanne 666607, Lausanne, Switzerland; 2 Institute of Microbiology, Lausanne University Hospital and University of Lausanne 536517, Lausanne, Switzerland; Washington University in St. Louis, St. Louis, Missouri, USA

**Keywords:** *Legionella*, water analysis, ISO 11731-2017

## Abstract

Legionnaires’ disease is a severe pneumonia caused by inhalation of *Legionella*-contaminated aerosols. The prevention of *Legionella* relies on water disinfection and on accurate water testing to assess the effectiveness of these interventions. The norm ISO 11731:2017 provides multiple culture-based methods for detecting *Legionella* in water and recommends taking into account the most unfavorable result. This study evaluated six ISO-standardized techniques, including variations in media, concentration, and pre-treatments (heat or acid), applied to 276 hospital hot water samples. *Legionella* was detected in 120 samples, with substantial variability across methods. The most sensitive single technique (76%) was concentration and elution with membrane filter on GVPC medium after heat treatment. Combinations of three techniques yielded a higher detection rate (98%), while using all six techniques detected all positive samples. Techniques involving concentration and elution proved superior to direct plating or simple filtration. Heat treatment enhanced detection more effectively than acid treatment, especially in samples with interfering flora. GVPC medium consistently outperformed BCYE. The study supports using a strategic combination of methods, particularly those involving membrane filtration and GVPC medium with heat treatment, to optimize *Legionella* detection in healthcare water systems.

**IMPORTANCE:**

This study is of critical importance as it provides a comprehensive evaluation of the sensitivity of various ISO 11731:2017-standardized methods for detecting Legionella in hospital water systems—a key concern in infection prevention. Given the high variability in recovery rates across different techniques and the limitations posed by adjacent flora, the study offers valuable insights into optimizing laboratory protocols. By analyzing 276 water samples using six techniques and their combinations, the researchers highlight the superiority of methods involving concentration, membrane filtration, and heat treatment—particularly those using GVPC medium. These findings not only support evidence-based recommendations for routine environmental surveillance in healthcare settings but also help laboratories balance accuracy, cost, and resource allocation. The study’s implications are especially relevant for institutions housing vulnerable populations, where early and reliable Legionella detection is essential to prevent potentially fatal outbreaks of Legionnaires’ disease.

## INTRODUCTION

Legionnaires’ disease (LD) is a severe and potentially fatal form of pneumonia caused by inhaling aerosolized water contaminated with *Legionella* bacteria from natural and artificial water systems. The disease can be either community-acquired or nosocomial, occurring in both sporadic and epidemic cases (1). The Swiss national guidelines for controlling and preventing legionellosis recommend annual or biannual water testing in healthcare institutions, with contamination thresholds based on *Legionella* concentration (cfu/L) (2). Therefore, laboratory protocols to accurately enumerate *Legionella* in water networks are essential to validate control measures and the effectiveness of disinfection interventions. The standard culture technique is the most used method for environmental surveillance of *Legionella*, estimating the bacterial count in water networks. One challenge in culturing is selecting a bacterial concentration technique that yields the highest recovery rate; for instance, membrane filtration has been shown to be superior to centrifugation. The second challenge is the inhibition of *Legionella* growth by the accompanying flora. To address this, three techniques are proposed (3): the addition of antibiotics to the culture media and/or the pretreatment of the sample with heat or acid.

The Swiss national guidelines, along with many other national guidelines, recommend using the International Organization for Standardization (ISO) norm 11731:2017 culturing method for *Legionella* enumeration across various water samples, including potable, industrial, waste, and natural water. This ISO standard provides three methods using selective culture media based on the water sample’s origin and characteristics: (i) direct plating, (ii) direct placement of membranes on agar media after filtration, and (iii) concentration and elution with membrane filters. Direct plating (0.1–0.5 mL) is used when a low concentration of interfering microorganisms and a high concentration of *Legionella* are expected. The other two methods are used when both interfering microorganisms and *Legionella* concentrations are low. However, all methods have highly variable recovery rates, complicating the accurate quantification of low *Legionella* concentrations. Even the most efficient recovery method can be quite inaccurate (4). Thus, ISO 11731:2017 recommends reporting the highest cfu/L obtained by any of the methods.

The aim of this study was to compare the sensitivity of six techniques (alone or combined) proposed in the ISO 11731:2017 standard and to determine which combination of these yields the highest *Legionella* contamination levels when analyzing hot water from hospital networks.

## RESULTS

A total of 276 water samples were analyzed by the six culture techniques (A, B1, B2, C1, C2, and D) and, overall, 120 were positive for *Legionella* (Table S1). Ninety-seven samples were contaminated with *L. pneumophila* alone, 15 with *L. anisa* alone, 7 with *L. pneumophila* and *L. anisa*, and 1 with *L. geestiana*. For further analysis, enumeration of *Legionella* was categorized into four groups: 0, < 100; 1, 100–999; 2, 1,000–9,999; 3, ≥ 10,000. These categories correspond to the threshold values defined in the Swiss recommendation for sanitary water in hospitals (2).

### Combination of techniques to reach the highest sensitivity

Relevant results from the number of positive samples obtained with a single technique or in combination are shown in Table 1. When results of the six techniques were combined, a total of 120 samples were found positive.

**TABLE 1:** Number of positive samples by combining different techniques^*a*^

Combination	Negative	No [10^2^–10^3^]	No [10^3^–10^4^]	No ≥10^4^	No POS	Sensitivity 1 (0 vs pos)	IC95% (0 vs pos)	Sensitivity 2 (<10^3^ vs ≥10^3^)	IC95% (<10^3^ vs ≥10^3^)
A + B1 + B2 + C1 + C2 + D	0	56	51	14	120	100%	[1–1]	100%	[1–1]
A + B1 + B2 + C2 + D	0	58	48	14	120	100%	[1–1]	97%	[0.95–0.98]
B2 + C2 + D	2	76	31	11	118	98%	[0.97–0.99]	66%	[0.61–0.69]
B1 + C2 + D	3	69	37	11	117	98%	[0.96–0.98]	75%	[0.71–0.78]
A + C2 + D	8	54	44	14	112	93%	[0.91–0.95]	91%	[0.87–0.93]
C2 + D	11	67	31	11	109	91%	[0.88–0.93]	66%	[0.61–0.69]
B2 + C2	12	71	29	8	108	90%	[0.87–0.92]	58%	[0.53–0.62]
A + B1 + C1	19	41	47	12	101	84%	[0.80–0.87]	94%	[0.91–0.95]
A + C2	23	44	41	12	97	81%	[0.77–0.84]	83%	[0.79–0.86]
C2	28	58	26	8	92	77%	[0.72–0.80]	53%	[0.48–0.57]
A + C1	34	32	42	12	86	72%	[0.67–0.75]	84%	[0.81–0.87]
C1	40	46	25	9	80	67%	[0.62–0.70]	53%	[0.48–0.57]
B2	42	52	22	4	78	65%	[0.60–0.69]	41%	[0.36–0.45]
B1	47	42	26	5	73	61%	[0.56–0.65]	48%	[0.43–0.52]
D	53	43	18	6	67	56%	[0.51–0.60]	38%	[0.33–0.41]
A	79	0	32	9	41	34%	[0.29–0.38]	64%	[0.59–0.68]

^*a*^ A, direct plating 1 mL, BCYE medium; B1, concentration and elution with membrane filter, BCYE medium, acid treatment; B2, concentration and elution with membrane filter, BCYE medium, heat treatment; C1, concentration and elution with membrane filter, GVPC medium, acid treatment; C2, concentration and elution with membrane filter, GVPC medium, heat treatment; and D, filtration 10 mL, GVPC medium. For each combination, the number of negative samples are indicated together with the number of positive stratified as follows [10^2^ to 10^3^] cfu/L, [10^3^ to 10^4^] cfu/L and ≥10^4^ cfu/L, the total number of positive, the sensitivity absence (0) versus presence of *Legionella* (pos), and the sensitivity to detect samples with ≥10^3^ cfu/L.

Using a single technique, concentration and elution with a membrane filter on GVPC medium combined with heat treatment (C2) yielded the highest number of positive samples (*N* = 92, corresponding to a sensitivity of 76%). In contrast, the least sensitive technique was direct inoculation of 1 mL (A), which resulted in only 41 positive samples (corresponding to a sensitivity of 34%). When three techniques were employed, the combinations B1 or B2 + C2 + D achieved the highest *Legionella* recovery rates, both at 98%. Using two techniques, the combination C2 + D demonstrated the best performance, achieving a *Legionella* recovery rate of 91%.

As in many guidelines the admitted level of contamination is 10^3^ cfu/L, the sensitivity to detect >10^3^ cfu/L was calculated (Table 1, Sensibility 2, 64 samples were positives with 10^3^ cfu/L). Interestingly, the combination of three techniques reaching the highest rate of detection was A + B1 + C1 (94%), whereas the highest rate with two techniques was obtained with A + C2 (83%) and A + C1 (84%).

The positivity of water samples at low bacterial counts (100–999 cfu/L), corresponding to 46.7% (*N* = 56) of the total positive samples, was determined mostly using techniques C2 (Table 1). On the other hand, high bacterial counts (>= 10,000 cfu/L) were mostly determined with techniques A, C1, and C2.

### Discordances between techniques

By comparing the recovery of *Legionella* of each technique two by two, we note that in all comparisons we observed positive samples with one technique that are negative by the other. Moreover, in all comparisons, the enumeration of at least one sample falls in two distinct categories separated by at least 1 Log_10_ (e.g., <100 and 1,000–9,999 cfu/L) (Table 2); this is especially the case when technique A was compared to technique D.

**TABLE 2:** Major discordances in the comparison of the recovery of *Legionella* of each technique two by two (number of samples for which the enumeration done by two techniques falls in two distinct categories)

	A	B1	B2	C1	C2
B1	19				
B2	12	6			
C1	21	13	7		
C2	19	9	5	1	
D	25	19	13	14	15

### Heat and acid treatment of the concentrates

After 3 days of incubation, 67 samples showed an important growth of adjacent flora after concentration and elution with membrane filter (techniques B and C), necessitating the heat and acid treatments of the concentrate. Thirty-five were positives among which 21 only after treatment. Most new positive samples (17/20) were recovered by heat treatment followed by inoculation on GVPC. Only 6/20 and 8/20 samples were positive after acid treatment followed by inoculation either on GVPC or BCYE, respectively, and 11/20 after heat treatment followed by inoculation on BCYE. Results in Table 1 also show that heat treatment of the concentrated sample gave a higher number of positives than acid treatment, this both on BCYE (B2) or GVPC (C2) plates.

### BCYE vs GVPC media

Focusing on the difference between the two media BCYE and GVPC, using the concentration and elution method, more positive samples were recovered on GVPC (92/120; techniques C1 and C2) than on BCYE (78/120; techniques B1 and B2). Moreover, among the 67 heat-treated samples, 26 (38%) were positive on BCYE and 31 (46%) on GVPC. Similarly, among the 67 acid treated samples, 17 (25%) were positive on BCYE and 12 (18%) on GVPC.

## DISCUSSION

In this study, potable water samples were analyzed using methods outlined in the ISO 11731:2017 standard to evaluate and compare *Legionella* recovery across the various techniques specified. We observed a great disparity in enumeration of *Legionella* according to the techniques used. As expected, the higher number of positive samples was obtained using all techniques in parallel. However, the workload involved is substantial, leading to an excessively high cost per analysis. It is essential to make strategic choices regarding the technique(s) to use, and our study provides the insight needed to make informed decisions.

Our results demonstrated that using a single technique (without treatment) recovers only up to 62% of positive samples. Furthermore, combining the two simple techniques—A (direct inoculation of 1 mL) and D (filtration of 10 mL)—yields a recovery rate of only 68%. This underscores the necessity of using the techniques B or C (concentration and elution using membrane filtration) for optimal *Legionella* recovery in hot water systems. Additionally, when adjacent flora is significant, our findings indicate that heat or acid treatment enhances the detection of previously undetected *Legionella*. The highest recovery of new positive samples was achieved with heat treatment followed by inoculation on GVPC medium, while acid treatment resulted in the lowest recovery rate. More importantly, water samples with low bacterial counts (100–1,000 cfu/L) accounted for nearly half (46.7%) of the positive results, with the majority of these detected using techniques B or C.

The BCYE medium has been newly introduced in the ISO 11731:2017 standard. This addition was questioned by several studies. Scaturro et al. showed that the GVPC medium was more efficient to detect *Legionella* than BCYE, especially at low bacterial counts (5). On the other hand, the study of Ditommaso et al. showed a better performance of BCYE compared to MWY, a selective medium similar to GVPC (6). The study of Jiménez Mayordomo also concluded that including BCYE significantly improves the recovery of *Legionella* in low-contaminated samples (7). Our study showed that, using the concentration and elution method, more positive samples were recovered on GVPC (73%) than on BCYE (43%). This is also corroborated by our results of enumeration after heat treatment, but not after acid treatment.

The primary limitation of our study is that, due to resource constraints, we were unable to test all the alternative techniques outlined in the ISO standard. For example, direct inoculation of 1 mL was conducted solely on BCYE medium, while filtration of 10 mL was performed only on GVPC medium. The second limitation of our study is that we filtered a volume of 10 mL for technique D, while other laboratories, such as in the study by De Giglio et al. (8), used a volume of 100 mL. We opted for 10 mL because preliminary tests revealed that half of the samples (14 out of 30) exhibited significant adjacent flora, likely inhibiting the growth of *Legionella*. Acid treatment of the filter might have reduced the adjacent flora; however, our results demonstrated limited *Legionella* recovery when this approach was applied. Despite these limitations, by comparing the results, we are confident that the strengths and weaknesses of each technique and medium were effectively documented, enabling informed decisions on the optimal protocol to adopt.

The choice of the techniques should be adapted to the needs, especially regarding the admitted level of contamination. The limits of 10^3^ cfu/L in public establishment vs <10^2^ in hospital wards with high-risk patients (intensive care units, onco-hematology units, …) might trigger the use of one combination or another (2).

All *Legionella* species are considered potentially pathogenic although the majority of human disease is caused by *L. pneumophila*; non-pneumophila species account for only about 2%–7% of legionellosis cases and predominantly occur in immunocompromised individuals. *Legionella anisa* is one of the most frequently isolated non-pneumophila species in environmental samples, such as cooling towers and hospital plumbing systems. Although its pathogenicity is considered low, it has been implicated in rare clinical cases. *Legionella geestiana*, by contrast, is primarily known from environmental isolates and, at our knowledge, has not been associated with human disease to date. Thus, *L. anisa* and *L. geestiana* are environmental species, often found in natural and man-made water systems. Most national guidelines require to detect all *Legionella* species in healthcare water system. Their presence means the water system provides ecological conditions favorable to *Legionella* colonization (e.g., warm temperatures, stagnation zones, biofilm). Even if these species are less pathogenic, their survival suggests that *L. pneumophila* could also establish itself under similar conditions.

In conclusion, our results showed that a single technique is not sufficient for the analysis of water samples. A combination of techniques, comprising at least the technique of concentration and elution with membrane filter, is needed for the recovery of *Legionella*. In order to optimize our laboratory protocol, we decided to implement techniques C (concentration and elution with membrane filter followed by inoculation on GVPC together with heat treatment if necessary) and D (filtration of 10 mL with the filter placed directly on GVPC medium).

## MATERIALS AND METHODS

**Fig 1:**
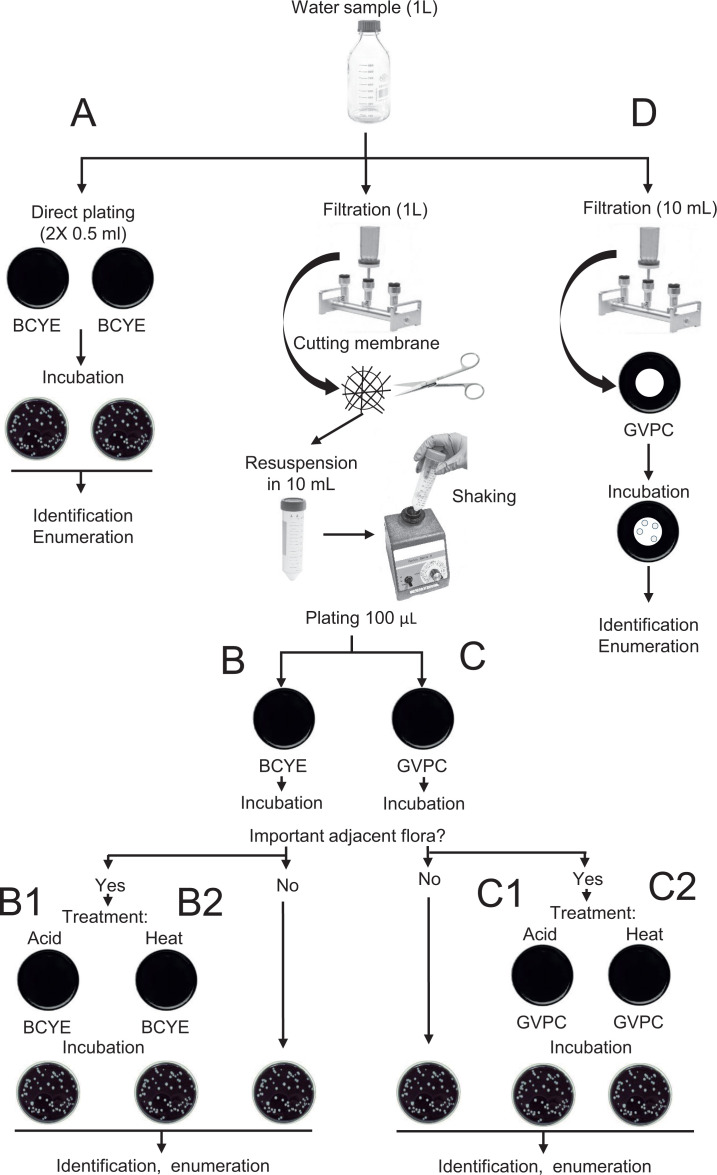
Illustration of the different techniques used in this study to analyze the recovery of *Legionella* in water samples.

Water samples were collected from hot water hospital networks. Sampling was performed at different sampling points (shower, faucet, boiler, etc.). According to the ISO 11731:2017, they were classified as belonging to the identified Matrix A, being water samples expected at low concentration of interfering microorganisms. For each sample, a volume of 1 L was collected and was processed with four techniques (Fig. 1).

Technique A consisted of the inoculation of 1 mL directly on buffered charcoal yeast extract agar plates (BCYE, bioMérieux, France). Two plates with 500 µL each were inoculated. For techniques B and C, 1 L of water was filtered using 0.22 µm polycarbonate membrane (Meissner, USA). The membrane was then aseptically cut into small pieces and placed in a screw cap sterile container with previously saved 10 mL of the water sample. Bacteria were resuspended by shaking vigorously for at least 2 min using a vortex mixer. One hundred microliters of this concentrated sample was plated on both Buffered Charcoal Yeast Extract agar plate (BCYE, bioMérieux, France) (technique B) and glycine, vancomycin, polymyxin B, cycloheximide agar plate (GVPC, bioMérieux, France) (technique C). Technique D consisted of filtering 10 mL of water on a 0.45 µm mixed cellulose esters membrane (Millipore, Germany), and the filter was placed directly on a GVPC agar plate. Each sample was analyzed once.

Incubation of all media was performed in aerobic conditions at 37°C in a humid environment. The plates were checked after 3, 5, and 7 days of incubation. At day 3 of incubation, if the growth of accompanying flora was too important on plates of techniques B and C, the concentrated sample was divided, and acid (techniques B1 and C1) and heat (techniques B2 and C2) treated according to ISO 11731:2017. One hundred microliters of each treated concentrate samples was plated on both BCYE and GVPC agars and incubated similar to untreated samples. After incubation, suspected colonies were identified directly, or after subculture, with the Maldi-Tof (Bruker, Germany). For *L. pneumophila* species, the serogroup was identified using the *Legionella* Latex Test (Thermo Fisher, Switzerland). *Legionella* colonies were counted, and the enumeration was reported to a number of cfu/L. For techniques B1, B2, C1, and C2, the highest number of cfu/L recovered either on the untreated or treated plate was considered. Note that the limits of detection are <10^3^ cfu/L for techniques A, B1, and C1; and <10^2^ cfu/L for techniques B2, C2, and D.

The sensitivity of each technique used alone or in combination was measured by taking as reference the identification of *Legionella* by at least one technique (Sensitivity 1) and the identification at the positivity threshold of 10^3^ (Sensitivity 2). These sensitivities were expressed with their 95% confidence interval.

The laboratory analyzing the samples is accredited ISO 17025 by the Swiss Accreditation Service (STS 0752).

## SUPPLEMENTAL MATERIAL

Table S1 aem.01147-25-s0001.xlsx Water samples: origin and results of enumeration of *Legionella* - raw data.

ASM does not own the copyrights to Supplemental Material that may be linked to, or accessed through, an article. The authors have granted ASM a non-exclusive, world-wide license to publish the Supplemental Material files. Please contact the corresponding author directly for reuse.
